# Effect of smoking-related COVID-19 risk messaging on smoking cessation in community smokers: A pragmatic randomized controlled trial

**DOI:** 10.18332/tid/163176

**Published:** 2023-06-14

**Authors:** Xue Weng, Tzu Tsun Luk, Yongda Socrates Wu, Sheng Zhi Zhao, Derek Yee Tak Cheung, Henry Sau Chai Tong, Vienna Wai Yin Lai, Tai Hing Lam, Man Ping Wang

**Affiliations:** 1 Institute of Advanced Studies in Humanities and Social Sciences, Beijing Normal University, Zhuhai, China; 2 School of Nursing, The University of Hong Kong, Hong Kong SAR, China; 3 Hong Kong Council on Smoking and Health, Hong Kong SAR, China; 4 School of Public Health, The University of Hong Kong, Hong Kong SAR, China

**Keywords:** smoking cessation, health communication, mobile health, COVID-19

## Abstract

**INTRODUCTION:**

Observational and experimental studies have suggested that messaging on smoking-related COVID-19 risk may promote smoking abstinence, but evidence from randomized clinical trials (RCTs) is lacking.

**METHODS:**

This was a pragmatic RCT in Hong Kong, China, to compare the effectiveness of communicating smoking-related COVID-19 risk with generic cessation support on abstinence. Both groups received brief cessation advice at baseline. The intervention group received messaging on smoking-related COVID-19 risk and cessation support via instant messaging for three months (16 messages in total), which highlighted the increased risk of severe COVID-19 and deaths, and potentially higher risk of viral exposure (e.g. due to mask removal) for smokers. The control group received generic text messaging support for three months (16 messages). The primary outcomes were biochemically validated 7-day point prevalence abstinence (PPA) at 3 and 6 months. Intention to treat analyses was used.

**RESULTS:**

Between 13 June and 30 October 2020, 1166 participants were randomly assigned to an intervention (n=583) or control (n=583) group. By intention-to-treat, validated 7-day PPA did not significantly differ between the intervention and control groups at three months (9.6% and 11.8%, relative risk, RR=0.81; 95% CI: 0.58–1.13, p=0.22) or six months (9.3% and 11.7%, RR=0.79; 95% CI: 0.57–1.11, p=0.18). A higher perceived severity of COVID-19 in smokers at baseline was associated with a greater validated 7-day PPA at six months, and a marginally significant intervention effect on changes in perceived severity from baseline through 6 months was found (p for group × time interaction = 0.08).

**CONCLUSIONS:**

Communicating smoking-related COVID-19 risk via instant messaging was not more effective in increasing smoking abstinence than generic cessation support.

**TRIAL REGISTRATION:**

The study is registered on ClinicalTrials.gov **Identifier:** NCT04399967

## INTRODUCTION

Systematic reviews and meta-analyses of observational studies have shown that current smokers have a higher risk of severe COVID-19 and death^1-3^. A systematic review of Mendelian randomization studies also showed that smoking traits (smoking initiation, smoking heaviness, lifetime smoking index) were associated with increased COVID-19 hospitalization, severity and mortality^4^. A potential mechanism is the upregulation of angiotensin converting enzyme-2 receptors (the adhesion site of SARS-CoV-2) in the respiratory tract due to cigarette smoke exposure^5^. Mask removal and the hand-to-mouth action of smoking may also predispose smokers to viral exposure^6^. The World Health Organization has recommended smoking cessation to reduce the burden of COVID-19^7^.

Messages regarding the risk of smoking on COVID-19 susceptibility and severity may influence smoking and quitting behaviors. Unverified claims that smoking can protect against COVID-19 have been widely disseminated, especially during the early phase of the pandemic^8^. Our population-based survey in Hong Kong found that exposure to such misinformation was associated with increased tobacco use^9^. On the other hand, observational studies in the US and UK showed that a higher perceived risk of COVID-19 related to smoking was associated with increased cessation-related behaviors (e.g. smoking reduction, quit attempts, and intentions)^10,11^, also shown in our study in Hong Kong^12^. Several online experiments have found that exposure to messaging on smoking-related COVID-19 risk can increase smokers’ motivations to quit^13,14^, which may be mediated by fear arousal^15^. Nevertheless, our PubMed search (until March 2023) using the keywords ‘risk communication’, ‘health communication’, ‘health warning’, ‘messaging’, ‘COVID-19’, and ‘smoking’ did not identify any randomized clinical trial (RCT) testing the effectiveness of such messaging on smoking abstinence in a real-world setting.

Our previous trial showed the effectiveness of instant mobile messaging in promoting smoking cessation^16^. Our subsequent trial showed the feasibility of using instant mobile messaging to deliver cessation support amid the COVID-19 pandemic^17^, in which access to in-person cessation services might be constrained. The current study compared the effectiveness of communicating smoking-related COVID-19 risk using instant mobile messaging with generic cessation support via text messaging to increase abstinence in community smokers.

## METHODS

### Trial design

This parallel, 2-arm, pragmatic RCT was conducted under the Hong Kong Council on Smoking and Health Quit-to-Win Contest^16,18-21^ in Hong Kong, China. Recruitment took place amid the 3rd wave of the COVID-19 pandemic, with approximately 4200 confirmed cases and 100 deaths in a population of approximately 7.5 million during the period of the recruitment (13 June to 30 October 2020, Supplementary file Figure S1)^22^. Stringent preventive measures were implemented, including mandatory masking and the restriction of public gatherings to two people, but without a city-wide lockdown^23^. The compliance rate of mask-wearing was nearly 100%. The Institutional Review Board of the University of Hong Kong/Hospital Authority Hong Kong West Cluster approved the protocol. The trial has been registered with ClinicalTrials.gov. The results are reported according to the Consolidated Standards of Reporting Trials (CONSORT-EHEALTH) reporting guidelines.

### Recruitment and participants

Participants were recruited from smoking hotspots in all 18 districts of Hong Kong or using online platforms. A total of 55 recruitment activities (booths in shopping malls and public areas) were organized at 47 community sites. University students and volunteers (n=110) from nongovernmental organizations attended a full-day online workshop and were trained as smoking cessation advisors. Similar to previous trials^16,18-21^, trained smoking cessation advisors proactively approached, screened and recruited smokers at smoking hotspots using the ‘foot-in-the-door’ approach^24^. Compared with the pre-pandemic period, fewer smokers were observed in outdoor smoking hotspots amid the pandemic^6^. Therefore, online advertisements were also used to recruit smokers, who were screened by a cessation advisor through video calls (eMethods in Supplementary file).

Eligible participants were Hong Kong residents who were aged ≥18 years, smoked at least 1 tobacco stick [cigarette or heated tobacco product (HTP)] per day or used e-cigarettes daily in the past 3 months, which was verified by a salivary cotinine level of ≥30 ng/mL, who was fluent in Cantonese, owned a smartphone with an instant messaging application installed, and had the intention to quit or reduce smoking. The exclusion criteria were participating in other smoking cessation programs or having physical or cognitive difficulties in communication. All eligible smokers willing to participate signed a written informed consent form.

### Interventions

All participants received brief cessation advice at baseline guided by the AWARD (Ask, Warn, Advise, Refer and Do-it-again) model, which was modified from the 5As (Ask, Advise, Assess, Assist, Arrange) model for use in the community settings, as in our previous trials conducted under the Quit-to-Win Contests^16,18-21^. Smoking cessation advisors asked the smokers about their smoking history (Ask), warned them about the hazards of smoking using a health warning leaflet (Warn), advised them to quit or reduce smoking as soon as possible (Advice), encouraged them to use a cessation service (Refer), and repeated the above steps if they continued to smoke or had relapsed at telephone follow-up sessions (Do-it-again).

Participants in the control group received a generic health warning leaflet that included shocking pictures of diseases caused by smoking, such as coronary heart disease, stroke, and lung cancer (Supplementary file Figure S2). They also received regular text messages (i.e. participants not able to respond to the messages) with generic cessation advice for three months from baseline, with a tapering schedule from twice a week in the first month to once a week in the following two months (16 messages in total). The messages included advice on smoking hazards and quitting benefits, strategies for quitting and coping, psychosocial support, and encouragement to initiate a quit attempt.

Participants in the intervention group received a health warning leaflet that highlighted the smoking-related COVID-19 risk (Supplementary file Figure S3). This included emphasizing the risk of respiratory infection, impaired immunity and COVID-19 complications in smokers (1.4-fold to 2.4-fold increased risk of requiring intensive care, using ventilators, and death)^25^, as well as the higher risk of viral exposure because of mask removal, hand-to-mouth action of smoking, clustering of smokers, and secondhand smoke. They also received a combination of regular messages and chat-based cessation support for three months via WhatsApp, an instant messaging application. The schedule of regular messages was the same as that in the control group (16 messages in total). The contents were similar to those of the control group, except the messages on smoking hazards focused on smoking-related COVID-19 risk. These messages were adopted from tweets and Facebook posts from the WHO, Centre for Health Protection of the Hong Kong Government, and the University of Hong Kong (Supplementary file Figure S4). Similar to our previous trials on mobile instant messaging support^16,17,26^, the participants could respond to the messages and initiate conversations with a research nurse to receive cessation coaching in real-time during office hours (9 a.m. to 6 p.m., Monday to Friday). Behavioral change techniques (e.g. eliciting and answering questions, providing feedback on current behaviors, boosting motivation and self-efficacy)^27^ were used based on the needs of the participants. An experienced doctoral-level researcher monitored the coaching process and discussed it with the research nurse when necessary.

### Randomization and blinding

A computer-generated randomization list was produced by an independent statistician using a randomized blocking schema (2, 4, or 6). Participants were individually assigned at random to groups with a 1:1 allocation ratio. Blinding the interventionists and participants was not possible because of the nature of the intervention. Outcome assessors and statistical analysts remained masked until the prespecified analyses were completed.

### Outcomes

Data were collected by questionnaire at baseline and telephone interviews at 1, 2, 3, and 6 months after intervention initiation. The primary outcome was biochemically verified 7-day PPA at three months (end of treatment) and six months after treatment initiation, verified by salivary cotinine concentrations of <30 ng/mL or exhaled carbon monoxide concentrations of <4 ppm. Research staff visited or video-called participants who reported having abstained from smoking for at least 7 days at 3 and 6 months for a biochemical validation (eMethods in Supplementary file)^28^. Secondary outcomes included self-reported 7-day point-prevalence abstinence (PPA), smoking reduction defined by a reduction of at least 50% in daily cigarette consumption compared with that at baseline, and intervention engagement defined as having read the instant messages or text messages in the respective intervention condition (none/some/all).

At baseline and the follow-up sessions, perceived susceptibility (i.e. smoking increased the risk of COVID-19 infection) and severity (i.e. smoking increased the severity of COVID-19 infection) of COVID-19 infection due to smoking were assessed on a scale of 0 to 10, with higher scores indicating greater perceived susceptibility/severity. We also assessed participants’ COVID-19-related information exposure (‘Have you ever received information on the link between smoking and COVID-19?’, yes vs no). Process evaluations were conducted by assessing the perceived appropriateness of the messages (yes vs no) and their usefulness in increasing the motivation to quit and quit attempt, each on a scale of 0 (not helpful at all) to 4 (very useful). Participants in the intervention group were asked if they had engaged in online conversations with the research nurse and the reasons for not doing so. Those who did engage in online conversations rated their satisfaction on a scale of 0 (not satisfied at all) to 10 (very satisfied).

### Statistical analysis

A meta-analysis of mobile health interventions showed an effect size [risk ratio (RR)] of 1.83 on biochemically verified 7-day PPA at six months^29^. Based on our prior studies^12,19^, we conservatively assumed an additional 10% relative increase in the intervention effect due to messaging on smoking-related COVID-19 risk; the anticipated biochemically verified PPA was 10.1% in the intervention group and 5.0% in the control group. With a type I error of 0.05, a power of 80% and an allocation ratio of 1:1, 421 participants were needed per group. Accounting for a retention rate of 75%, a total of 1122 participants was needed.

We used intent-to-treat analyses, and participants with missing outcomes were imputed as smokers with no change in tobacco consumption. Poisson regression models with robust variance estimators were used to calculate the RRs of the intervention effect on the primary and secondary outcomes. The associations of baseline perceived susceptibility/severity with 6-month biochemically verified PPA were examined, adjusting for sociodemographic and known determinants of quitting^30^. Sensitivity analyses were conducted for the abstinence outcomes. First, we used complete case analyses by excluding participants with missing outcomes. Second, we used multiple imputation by chained equations to impute missing abstinence outcomes, including study group, sex, age, education level, household income, previous quit attempts, cigarette dependence, and intention to quit. Rubin’s rule was used to pool the estimates from 50 imputed datasets^31^. Mixed-effect models were used to examine the differences in changes in the perceived susceptibility and severity of COVID-19 due to smoking from baseline through 6 months. All analyses were conducted using Stata V.15.1 (StataCorp, Texas, USA), with p<0.05 indicating statistical significance.

## RESULTS

### Participant characteristics

**Figure 1:**
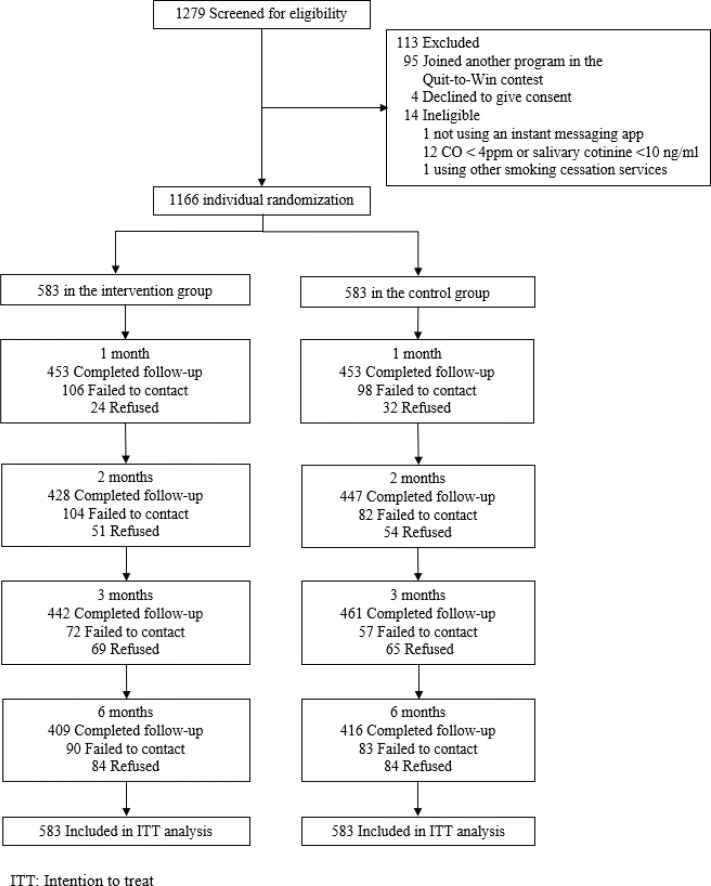
Study flow diagram

Figure 1 shows that 1166 participants were randomized to the intervention (n=583) or control group (n=583). The participants were mostly male (79.1%), aged 30–59 years (64.2%), married or cohabiting (51.4%), and had completed secondary education (62.4%); 76.4% of the participants were recruited in smoking hotspots and 23.6% via online platforms. Participants smoked an average of 13.5 cigarettes per day, 71.5% had previous quit attempts, and 50.3% were ready to quit within the next 30 days. Table 1 shows that the baseline characteristics and smoking profiles were similar between the groups. The retention rates were 71–78% at the follow-up sessions and did not differ by group (p=0.21–84).

**Table 1:** Baseline demographic and smoking-related characteristics (N=1166)

Characteristics	Intervention group (N=583) n (%)	Control group (N=583) n (%)
**Recruitment methods**		
Onsite	447 (76.7)	444 (76.2)
Online	136 (23.3)	139 (23.8)
**Sex**		
Male	452 (77.5)	470 (80.6)
Female	131 (22.5)	113 (19.4)
**Age** (years)		
≤29	118 (20.5)	143 (24.7)
30–59	377 (65.5)	365 (63.0)
≥60	81 (14.1)	71 (12.3)
**Marital status**		
Single	225 (38.9)	254 (44.2)
Married/cohabited	311 (53.7)	282 (49.0)
Divorced/separated/widowed	43 (7.4)	39 (6.8)
**Education level**		
Primary or lower	35 (6.3)	27 (4.7)
Secondary	354 (63.3)	350 (61.4)
Tertiary	170 (30.4)	193 (33.9)
**Monthly household income** (HK$)		
<25000	243 (43.5)	220 (38.9)
25000–59999	240 (43.0)	267 (47.3)
>60000	75 (13.4)	78 (13.8)
**Daily use of tobacco products**		
Cigarettes	508 (87.1)	518 (88.9)
HTPs	18 (3.1)	10 (1.7)
E-cigarettes	14 (2.4)	14 (2.4)
≥ 2 tobacco products	43 (7.4)	41 (7.0)
Daily cigarette consumption, mean ± SD	13.4 ± 9.8	13.5 ± 9.3
**Time to first cigarette after waking,** (min)		
≤30	347 (59.7)	352 (60.5)
>30	234 (40.3)	230 (39.5)
**Age started smoking** (years)		
≤17	268 (46.0)	255 (43.9)
18–25	277 (47.5)	293 (50.4)
≥26	38 (6.5)	33 (5.7)
**Past quit attempts**		
No	170 (29.2)	161 (27.8)
Yes	413 (70.8)	418 (72.2)
**Readiness to quit** (days)		
≤30	284 (49.0)	300 (51.6)
>30	296 (51.0)	281 (48.4)
**Perception of quitting,** mean ± SD		
Importance	7.17 ± 2.4	7.21 ± 2.4
Confidence	5.69 ± 2.5	5.90 ± 2.5
Difficulty	6.87 ± 2.7	6.77 ± 2.9

HTP: heated tobacco product. Sample size varied because of missing data. HK$7.8 about US$1.0.

### Smoking cessation outcomes

Table 2 shows that the primary outcome of biochemically verified PPA was not significantly different between the intervention and control groups at 3 months (9.6% vs 11.8%, RR=0.81; 95% CI: 0.58–1.13, p=0.22) or 6 months (9.3% vs 11.7%, RR=0.79; 95% CI: 0.57–1.11, p=0.18). For secondary outcomes, self-reported 7-day PPA was significantly lower in the intervention group than in the control group at 3 months (14.8% vs 21.8%, RR=0.68; 95% CI: 0.53–0.87, p=0.002) but not at 6 months (15.6% vs 19.4%, RR=0.81; 95% CI: 0.63–1.04, p=0.09). Both groups reported similar smoking reduction rates. Sensitivity analyses based on the complete case and multiple imputations showed similar results (Supplementary file Table S1). Multivariable regression showed that a higher perceived severity (RR=1.07; 95% CI: 1.00–1.13, p=0.04) but not perceived susceptibility (RR=1.01; 95% CI: 0.95–1.07, p=0.82) at baseline was associated with biochemically verified PPA at 6 months (Supplementary file Table S2).

**Table 2:** Primary and secondary outcomes by ITT analysis (N=1166)

*Outcomes*	*Intervention group (N=583) n (%)*	*Control group (N=583) n (%)*	*RR (95% CI)*	*p*
**Primary outcomes**				
**Biochemically validated 7-day PPA**				
3-month	56 (9.6)	69 (11.8)	0.81 (0.58–1.13)	0.22
6-month	54 (9.3)	68 (11.7)	0.79 (0.57–1.11)	0.18
**Secondary outcomes**				
**Self-reported 7-day PPA**				
1-month	48 (8.2)	73 (12.5)	0.66 (0.47–0.93)	**0.017**
2-month	65 (11.1)	91 (15.6)	0.71 (0.53–0.96)	**0.026**
3-month	86 (14.8)	127 (21.8)	0.68 (0.53–0.87)	**0.002**
6-month	91 (15.6)	113 (19.4)	0.81 (0.63–1.04)	0.09
**Smoking reduction^a^**				
1-month	128 (22.0)	131 (22.5)	0.98 (0.79–1.21)	0.83
2-month	123 (21.1)	128 (22.0)	0.96 (0.77–1.20)	0.72
3-month	120 (20.6)	108 (18.5)	1.11 (0.88–1.40)	0.38
6-month	119 (20.4)	102 (17.5)	1.17 (0.92–1.48)	0.21

ITT: intention-to-treat. RR: risk ratio. PPA: point prevalence of abstinence.

a Quitting not included as reduction but included in denominators.

### Change in perceived susceptibility and severity

Table 3 shows that the perceived susceptibility and severity of COVID-19 infection were not significantly different between the groups at baseline and at the follow-up sessions, except perceived severity was higher in the intervention group than in the control group at 2 months (6.2 vs 5.9; p=0.045). Exploratory analyses using mixed-effect models showed no significant intervention effect on the changes in perceived susceptibility (p for group × time interaction = 0.19) but a marginally significant effect on perceived severity (p for group × time interaction = 0.08) from baseline through 6 months.

**Table 3:** Changes in perceived susceptibility and severity^a^ of COVID-19 due to smoking by groups (N=1166)

	*Intervention group (N=583) Mean (SD)*	*Control group (N=583) Mean (SD)*	*Mean difference*	*p for mean difference*	*p for group × time interaction*
**Perceived susceptibility**					
Baseline	7.7 (3.1)	7.7 (3.1)	0.004	0.98	0.19
1-month	7.2 (3.1)	7.3 (3.1)	-0.80	0.67	
2-month	7.2 (3.0)	7.2 (3.0)	-0.05	0.80	
3-month	7.6 (2.7)	7.4 (2.8)	0.16	0.40	
6-month	7.8 (2.8)	7.5 (2.9)	0.32	0.10	
**Perceived severity**					
Baseline	6.1 (3.3)	6.2 (3.3)	-0.04	0.81	0.08
1-month	6.3 (2.8)	6.3 (3.0)	0.12	0.47	
2-month	6.2 (2.6)	5.9 (2.9)	0.32	0.045	
3-month	6.0 (2.2)	6.1 (2.5)	-0.42	0.79	
6-month	5.8 (2.6)	5.9 (2.8)	-0.08	0.62	

a Assessed on a scale of 0 to 10, with higher scores indicating greater perceived susceptibility or severity.

### Intervention engagement and ratings

Table 4 shows that the proportion of participants who had read the instant/text messages was similar between the two groups. Biochemically verified 7-day PPA at 6 months was higher in engaged subgroups (e.g. read messages, had conversations with research nurse, and read messages and had conversations) than non-engaged participants (Supplementary file Table S3). The perceived appropriateness of the messages was high overall, although it was significantly lower in the intervention group than in the control group (86.5% vs 90.6%, p=0.04). Similarly, the perceived usefulness of the messages in increasing motivation to quit (2.3 vs 2.4, p=0.02) and quit attempts (2.2 vs 2.4, p=0.02) was significantly lower in the intervention group.

**Table 4:** Intervention engagement and ratings (N=1166)

	*Intervention group (N=583) n (%)*	*Control group (N=583) n (%)*	*p*
**Intervention engagement**			
Have ever read instant/text messages			0.11
None^a^	94 (16.1)	113 (19.4)	
Some	142 (24.4)	116 (19.9)	
All	347 (59.5)	354 (60.7)	
**Rating of the messages**			
The intensity of messages was appropriate (yes vs no)	423 (86.5)	426 (90.6)	**0.04**
The contents of messages increased motivation to quit^b^, mean ± SD	2.3 ± 1.0	2.4 ± 1.1	**0.02**
The contents of messages increased quit attempts, mean ± SD	2.2 ± 1.0	2.4 ± 1.1	**0.01**
**Exposure to information on COVID-19 and smoking**			
Baseline	192 (33.3)	207 (36.1)	0.32
1-month (cumulative)	372 (64.2)	309 (53.4)	**<0.001**
2-month (cumulative)	426 (73.6)	388 (66.7)	**0.01**
3-month (cumulative)	482 (83.2)	448 (77.0)	**0.008**
6-month (cumulative)	489 (84.5)	470 (80.8)	0.09

a Participants with missing data were counted as ‘None’ in the analysis.

b Score: 0–4, higher scores indicating higher usefulness of messages.

In the intervention group, 49.4% (n=288) of the participants had conversations with the research nurse, with a mean satisfaction score of 8.5 (SD=1.9) on a scale of 0 to 10. Being ‘too busy’ (49.5%) was the most common reason for not conversing with the research nurse, followed by ‘don't want to talk about cessation-related topics online’ (32.9%), ‘not interested’ (19.7%), and ‘not useful’ (5.4%).

## DISCUSSION

This is the first RCT testing the effectiveness of messaging on smoking-related COVID-19 risk in increasing smoking abstinence in a real-world setting. Contrary to our expectations, communicating smoking-related COVID-19 risk via instant mobile messaging did not increase biochemically verified 7-day PPA at 3 and 6 months when compared with generic cessation support via text messaging amid the COVID-19 pandemic. However, the 6-month verified PPA (approximately 10%) in both groups was much higher than that of the intervention groups in our previous RCTs conducted under the Quit-to-Win contests before the COVID-19 pandemic (ranging from 5.6% to 9.0%)^16,18-21^. A 2019 Cochrane review of 13 RCTs showed that text messaging-based behavioral support was effective in promoting abstinence^29^. Our findings indicate that communicating messages on smoking-related COVID-19 risk with a research nurse via instant messaging did not confer additional benefits.

The intervention was designed to promote cessation by increasing the perceived smoking-related risk of COVID-19, which has been found to be associated with intermediate cessation outcomes such as the motivation to quit in observational and experimental studies^10-14^. Our secondary analyses also showed that greater perceived severity of COVID-19 due to smoking was associated with 6-month verified PPA, adjusting for known determinants of successful cessation. However, we did not find a significant difference in changes in perceptions between the two groups, which may explain the null effect on abstinence. Subgroup analyses between online and face-to-face recruitment found no significant difference in abstinence, suggesting the similar null effects of interventions in both groups.

Several reasons may underlie the failure of the intervention to change smoking-related COVID-19 risk perception. Our survey conducted prior to the trial showed that most smokers did not think smoking increased COVID-19 susceptibility (72%) or severity (58%)^12^. However, our trial participants showed relatively high levels of perceived susceptibility and severity (mean scores = 7.7 and 6.2 out of 10, respectively) at baseline. This ‘ceiling effect’ might have constrained the room for increasing the risk perception level. Second, over 80% of the participants in both groups reported exposure to information on COVID-19 and smoking from any sources during the study period. The control group likely was exposed to such information from other sources, such as publicity by the local health authority and cessation services. Such contamination might have biased the intervention effect toward the null. Finally, participants’ ratings for the messages were significantly lower in the intervention group than in the control group, which suggests that messaging on smoking-related COVID-19 risk was less preferred compared to generic messages and cessation support. Prior qualitative studies on mobile messaging showed that smokers might not be receptive to messages that repeatedly emphasize the hazards of smoking (loss-framed messages)^32,33^. Further research is warranted to determine the optimal ‘dose’ to communicate smoking-related COVID-19 risk without demotivating smokers.

Studies on smokers’ affective responses to messages on smoking and COVID-19 also provide some insights into our null findings. An experiment showed that messages on smoking-related COVID-19 risk (vs traditional risk) elicited similar levels of reactions (e.g. attention, negative affect) and were perceived to be similarly effective in discouraging smoking^14^. Likewise, another experiment showed no difference in valence or arousal elicited by COVID-19 or traditional graphic health warnings on cigarette packaging^34^. The experiment also showed that smokers with lower delay discounting (i.e. the preference for smaller immediate rewards over larger but delayed rewards) were more likely to be aroused by COVID-19 warnings^34^. The much milder impact of COVID-19 in Hong Kong than in most other places worldwide might have led to a lower perceived benefit of cessation in reducing COVID-19 risk and, thus, the abstinence rate.

### Strengths and limitations

The strengths of the study include the randomized trial design conducted with a large cohort of smokers (n=1166) in a real-world setting with the use of an active comparison group. Another strength is the use of biochemically verified PPA as the outcome with a satisfactory participation rate (64%; 144/226) despite the difficulties of conducting in-person validation amid the pandemic.

This trial also had several limitations. First, despite a satisfactory retention rate of 71% at 6 months, given the minimal contact between the participants and researchers, non-response bias could not be excluded. However, our sensitivity analyses based on multiple imputations and complete cases yielded similar results to those of the main analyses. Second, as discussed, most participants in the control group were exposed to information on COVID-19-related smoking risks, which might reduce the intervention effect size, if any. However, given the real-world trial design, such contamination is difficult to control^35^. Third, the secondary outcome of intervention engagement (i.e. having read the instant messages or text messages) was based on self-report. Finally, our trial was conducted in Hong Kong, where the prevalence of smoking is low (9.5%), and smokers are predominantly male^36^. The generalizability of the findings to other places is unclear.

## CONCLUSIONS

Communicating smoking-related COVID-19 risk via instant messaging was not more effective in increasing smoking abstinence than generic cessation support via text messaging.

## Data Availability

The data supporting this research are available from the authors on reasonable request.
