# Supplementary material for: Effect of smoking-related COVID-19 risk messaging on smoking cessation in community smokers: A pragmatic randomized controlled trial
Source: Tob Induc Dis. 2023 Jun 14;21:77. doi: 10.18332/tid/163176 (PMC10266330; doi:10.18332/tid/163176)
Supplement: Supplementary file 1 [file TID-21-77-s1.pdf]

Supplementary appendix

eMethods

Figure S1 Number of confirmed COVID-19 cases in Hong Kong (Jan 23, 2020-April 30, 2021)

Figure S2 Generic health warning leaflet in the control group

Figure S3 COVID-19 warning leaflet in the intervention group

Figure S4 Examples of text and image messages in the intervention group

Table S1. Sensitivity analyses on primary and secondary outcomes

Table S2. Baseline predictors of validated abstinence at 6 months

eMethods

1. Details of video-call screening

Online registered smokers who were determined to be initially eligible were called to schedule an office visit with research staff. Smokers who were unable to attend office visits were asked to provide their mailing addresses. Smoking cessation advisors mailed screening packages (i.e., consent forms, questionnaires, and cotinine supplies) to the participants' homes and scheduled a video-call screening afterward. For video call cotinine test procedures, please refer to the "Details of biochemical validation" section below.

2. Details of biochemical validation

Participants who self-reported abstinence for more than 7 days at 3 and 6 months were invited for biochemical validation by salivary cotinine or expired carbon monoxide (CO). Biochemical tests were performed in person or remotely under the instruction of research staff. For participants who were unable to attend the in-person test, research staff mailed cotinine supplies to their home addresses and scheduled video-call validation tests when the supplies were received.

Saliva cotinine levels were measured using the Alere iScreen® Oral Fluid Device (OFD) Test (New Line Medical).[1] Participants were instructed to insert the cotton swab into their mouth and actively move it inside the mouth and tongue for at least 3 min. After the cotton swab was completely saturated with saliva, the cotton swab was inserted vertically into the cap and then placed horizontally for the 10-min processing time until a result indicating the participant's smoking status appeared on the result window. A negative iScreen® result (i.e., cotinine concentrations <30 ng/ml) was classified as biochemically verified abstinence. For participants who reported using nicotine replacement therapy (NRT), abstinence was biochemically verified by providing a breath carbon monoxide (CO) sample <4 ppm. Exhaled CO was assessed by a Smokerlyzer® (Bedfont Scientific Ltd). Participants were instructed to take a deep breath, hold it for 15 seconds, and exhale slowly into the mouthpiece of the Smokerlyzer®.

As an award of the "Quit to Win" contest, all participants who passed the biochemical validation were given a small cash price of HK$500 (≈US$64). Our previous "Quit to Win" contest found that such a small, abstinence-contingent cash incentive had no effect on smoking cessation outcomes.[2]

Supplementary results

Figure S1 Number of confirmed COVID-19 cases in Hong Kong (Jan 23, 2020-April 30, 2021)

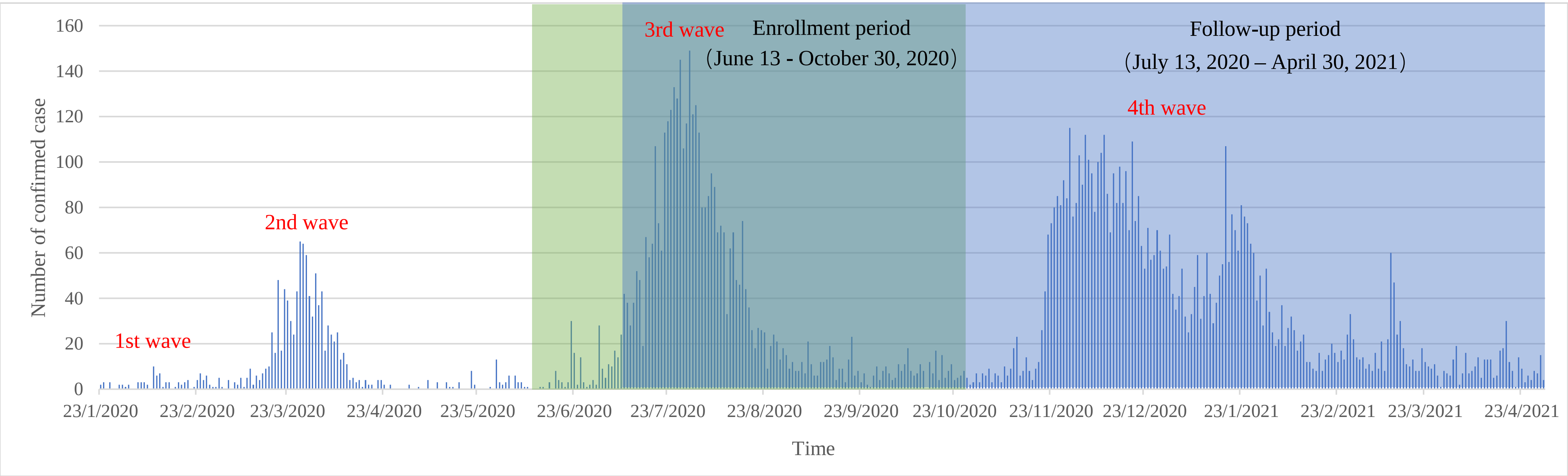

Data were adapted from the center for Health Protection.[3]

Figure S2 Generic health warning leaflet in the control group

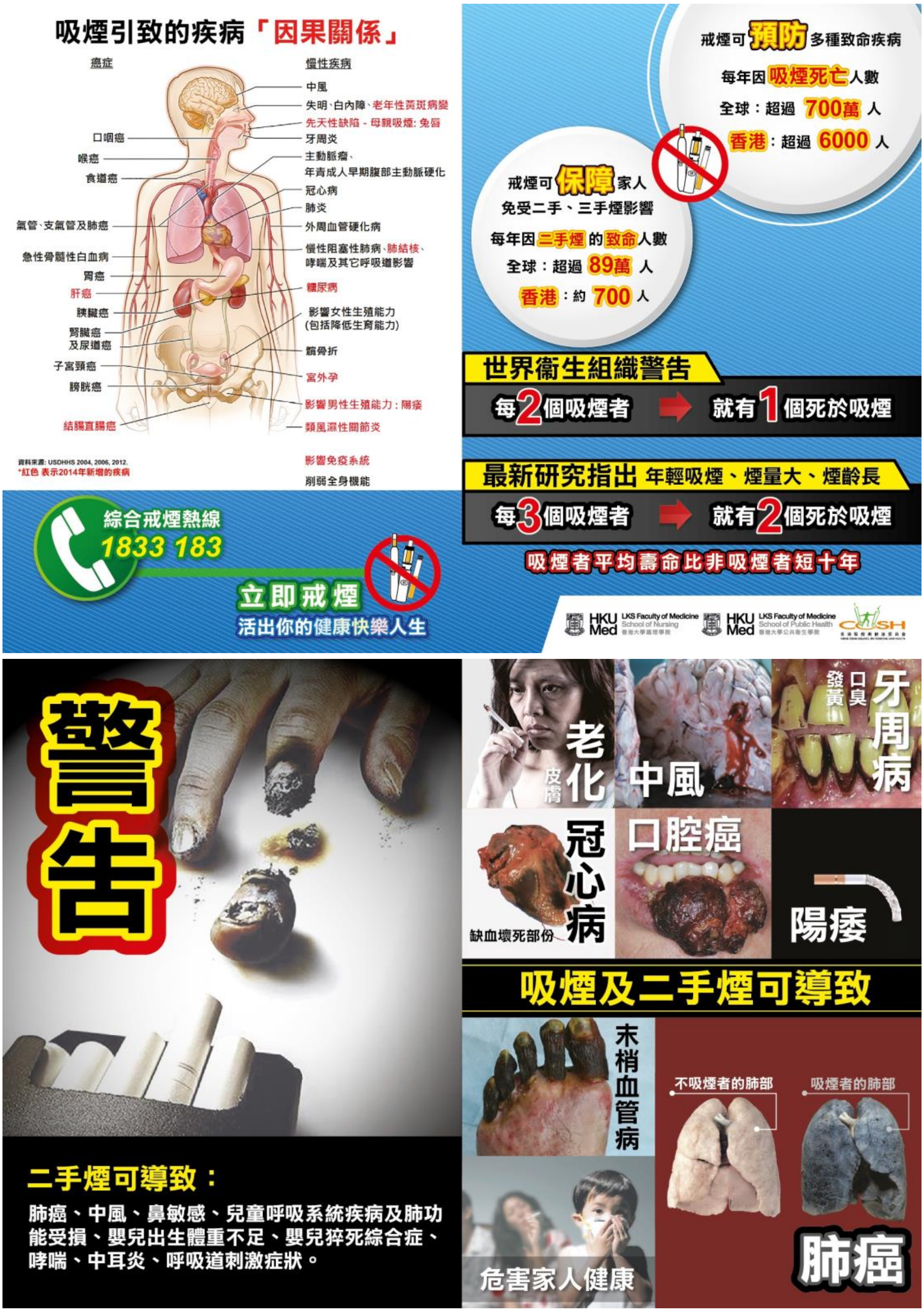

The content of the general health warning leaflet included the following: (1) the absolute risk of death due to smoking; (2) the whole list of diseases caused by active and second-hand smoking; (3) ten horrible pictorial warnings of health consequences of smoking and secondhand smoke; (4) the benefits of smoking cessation; and (5) simple messages to encourage participants to quit smoking and remind them to call the smoking cessation hotline 1833183.

Figure S3 COVID-19 warning leaflet in the intervention group

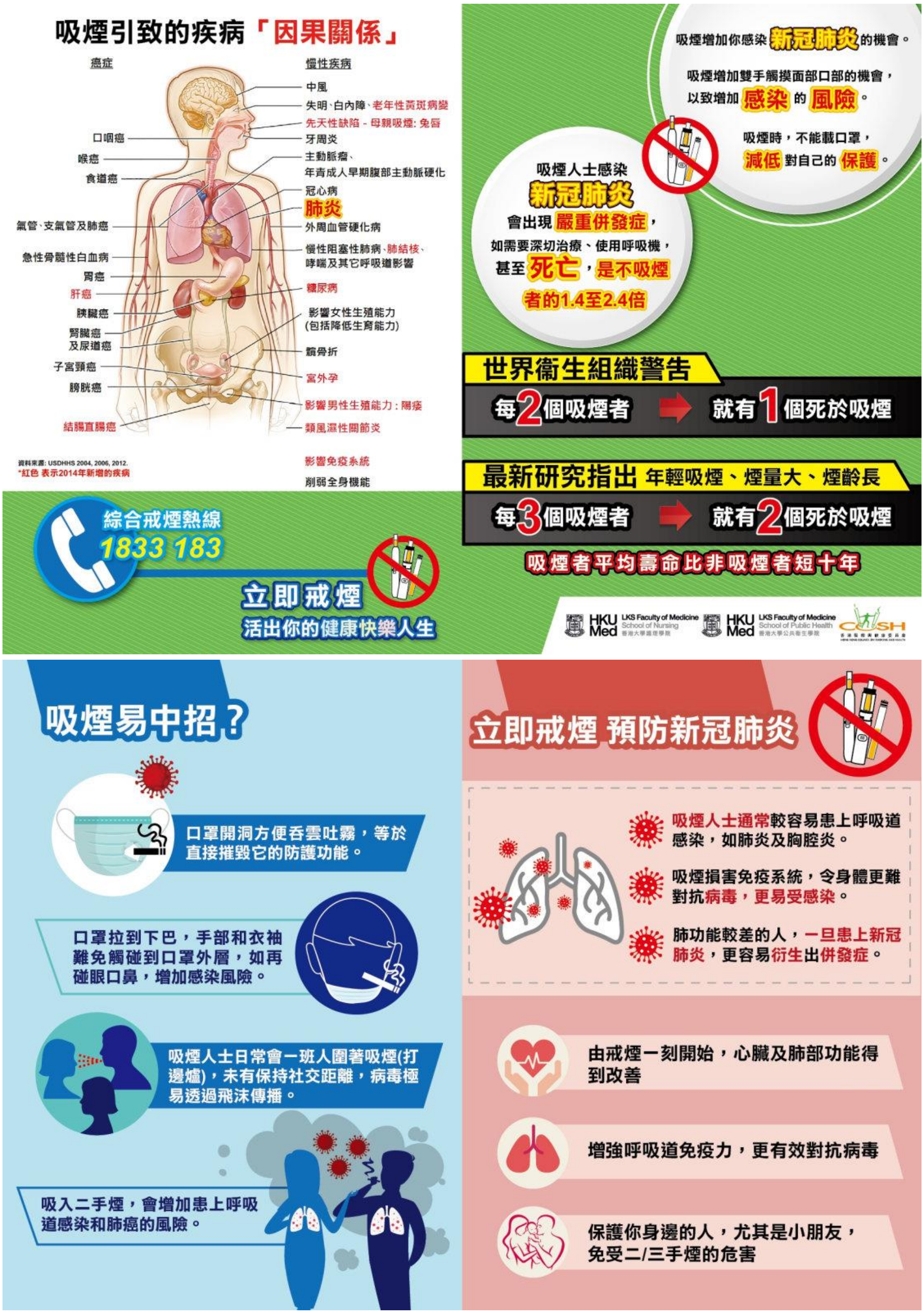

The content of the COVID-19 warning leaflet included the following: (1) the absolute risk of death due to smoking; (2) the whole list of diseases caused by active and second-hand smoking; (3) the risks of COVID-19 due to smoking (susceptibility, severity, hospitalization, and mortality); (4) the benefits of smoking cessation during the COVID-19 pandemic; and (5) simple messages to encourage participants to quit smoking and remind them to call the smoking cessation hotline 1833183.

Figure S4 Examples of text and image messages in the intervention group

<table>
<tr><th>Message types</th><th>Message contents</th></tr>
<tr><td>Severity of COVID-19 outcomes</td><td>(a) 最新研究顯示，新冠肺炎病例中，吸煙者感染且病情惡化的機率比非吸煙者高。不妨趁防疫期間戒咗佢😉，輕鬆又健康💪💪💪！
[Recent studies have shown that among COVID-19 patients, smokers are more likely than nonsmokers to be infected and to have more severe treatment outcomes. Quit smoking during the COVID-19 pandemic, be healthy and be relaxed.]
(b) 吸煙損害肺部和削弱免疫力。一旦感染新冠肺炎，年齡大嘅人，患有心臟病，呼吸系統疾病或有吸煙習慣嘅人，有更大機會患上重症😨
[Smoking damages the lungs and weakens the immune system. Once infected with COVID-19, those who are older, have heart disease or respiratory disease, or who are smokers have a higher chance of severe COVID-19 infection.]
(c) 吸煙者感染新冠病毒重症概率是不吸煙者的 1.91 倍😨 為保護自己同身邊嘅人，即刻戒煙啦！
[Smokers are 1.91 times more likely to have severe COVID-19 symptoms than nonsmokers. Quit smoking now to protect yourself and the people around you.]</td></tr>
<tr><td>Susceptibility to COVID-19 infection</td><td>Image messages adopted from tweets and Facebook posts from (a) WHO, (b) Centre for Health Protection of the Hong Kong Government, and (c) Li Ka Shing Faculty of Medicine of the University of Hong Kong
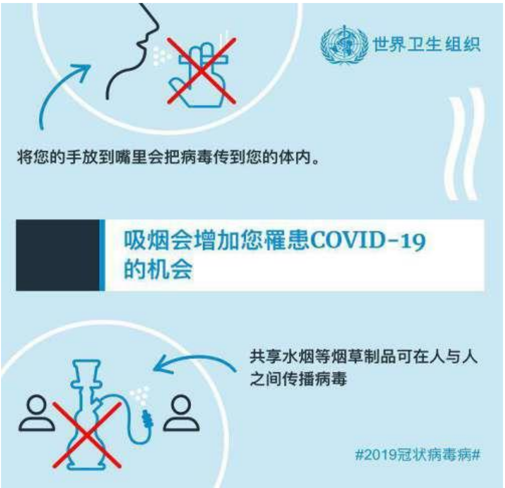

(a)
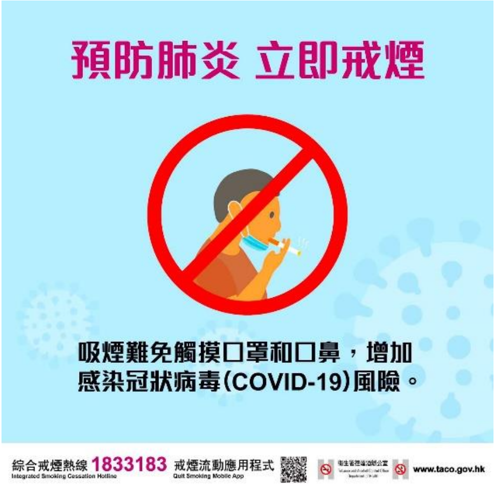

(b)
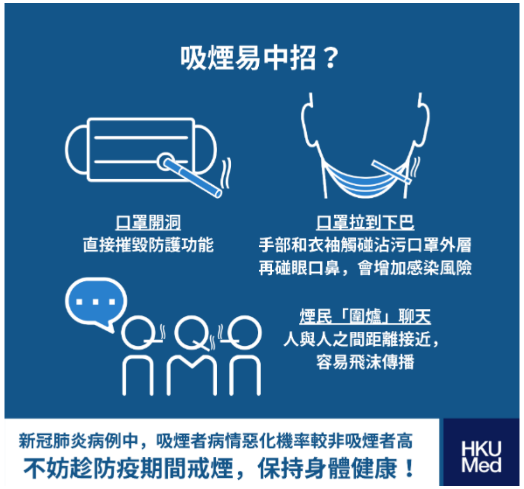

(c)</td></tr>
</table>

Table S1. Sensitivity analyses on primary and secondary outcomes

	Complete case		Multiple imputation	
	RR (95% CI)	*P* value	RR (95% CI)	*P* value
**Primary outcomes**				
Biochemically verified 7-day PPA				
3-month	0.85 (0.61-1.17)	0.32	0.86 (0.62-1.21)	0.40
6-month	0.81 (0.58-1.12)	0.21	0.82 (0.58-1.17)	0.27
**Secondary outcomes**				
Self-reported 7-day PPA				
1-month	0.66 (0.47-0.92)	0.02	0.67 (0.48-0.95)	0.02
2-month	0.74 (0.56-0.99)	0.046	0.76 (0.56-1.02)	0.07
3-month	0.71 (0.56-0.90)	0.005	0.73 (0.56-0.95)	0.02
6-month	0.82 (0.64-1.04)	0.10	0.85 (0.66-1.10)	0.23
Smoking reduction[a]				
1-month	0.98 (0.80-1.20)	0.83	1.00 (0.79-1.25)	0.97
2-month	1.00 (0.81-1.23)	0.99	0.99 (0.79-1.24)	0.94
3-month	1.16 (0.93-1.45)	0.20	1.20 (0.93-1.55)	0.16
6-month	1.19 (0.95-1.49)	0.14	1.18 (0.93-1.50)	0.18

RR: risk ratio; CI: confidence interval; PPA: point prevalence of abstinence.

[a] Quitting not included as reduction.

Table S2. Baseline predictors of 6 month biochemically verified PPA (N=1166)

Characteristics	Adjusted RR (95% CI) [a]	Adjusted RR (95% CI) [b]
Sex		
Male	Ref	Ref
Female	0.79 (0.50-1.27)	0.69 (0.42-1.12)
Age group, years		
⩽29	Ref	Ref
30-59	1.25 (0.86-1.83)	1.15 (0.78-1.71)
⩾60	1.04 (0.59-1.84)	0.84 (0.46-1.53)
Daily cigarette consumption, stick		
1-10	Ref	Ref
>11	0.55 (0.37-0.81)**	0.55 (0.37-0.82)**
Past quit attempt(s)		
No	Ref	Ref
Yes	1.54 (0.99-2.39)	1.21 (0.78-1.90)
Readiness to quit, days		
>30	Ref	Ref
⩽30	2.88 (1.92-4.34)***	2.44 (1.61-3.71)***
Recruitment methods		
Onsite	Ref	
Online	2.40 (1.68-3.44)***	1.96 (1.35-2.83)***
Perceived susceptibility of COVID-19 due to smoking	1.01 (0.95-1.07)	1.01 (0.95-1.07)
Perceived severity of COVID-19 due to smoking	1.08 (1.02-1.14)*	1.07 (1.00-1.13)*

PPA: point prevalence of abstinence, RR: risk ratio

[a] Adjusted for group.

[b] Adjusted for group and mutually adjusted.

* P<0.05, ** P <0.01, *** P <0.001

Table S3. Biochemically validated 7-day PPA at 6 months by intervention engagement

	Validated abstinence	Poisson regression	
	n (%)	Crude RR (95% CI)	Adjusted RR[b] (95% CI)
Intervention group			
Not engaged[a] (n=94)	0 (0.0)	-	-
Read some messages only (n=71)	0 (0.0)	-	-
Read some messages & had conversations (n=71)	3 (4.2)	Ref	Ref
Read all messages only (n=130)	4 (3.1)	0.73 (0.17-3.17)	0.63 (0.15-2.68)
Read all messages & had conversations (n=217)	47 (21.7)	5.13 (1.64-16.00)**	4.21 (1.33-13.30)*
Control group			
Not engaged[a] (n=113)	2 (1.8)	Ref	Ref
Read partial messages (n=116)	66 (14.0)	1.95 (0.36-10.40)	2.05 (0.39-10.70)
Read all messages (n=354)	68 (11.7)	9.90 (2.46-39.90)**	9.65 (2.48-37.50)**

* $P < 0.05$;** $P < 0.01$;*** $P < 0.001$.

Abbreviation: PPA: point prevalence of abstinence, RR: risk ratio; CI: confidence interval.

[a] Participants with missing data were counted as non-engagement in the analysis.

[b] Adjusted for sex, age, past quit attempts, and readiness to quit at baseline.

References:

1. Moore MR, Mason MJ, Brown AR, Garcia CM, Seibers AD, Stephens CJ. Remote biochemical verification of tobacco use: Reducing costs and improving methodological rigor with mailed oral cotinine swabs. *Addict Behav.* 2018;87:151-154.
2. Cheung YTD, Wang MP, Li HCW, et al. Effectiveness of a small cash incentive on abstinence and use of cessation aids for adult smokers: a randomized controlled trial. *Addictive Behaviors.* 2017;66:17-25.
3. Hong Kong Centre for Health Protection. Archives of Latest situation of cases of COVID-19. 2022; https://www.chp.gov.hk/en/features/102997.html. Accessed May 30, 2022.

© 2023 Weng X. et al.
